# Knowledge, attitude and practice related to rabies among residents of Amhara region, Ethiopia

**DOI:** 10.1016/j.heliyon.2022.e11366

**Published:** 2022-11-03

**Authors:** Adane Bahiru, Wassie Molla, Liuel Yizengaw, Sefinew Alemu Mekonnen, Wudu Temesgen Jemberu

**Affiliations:** a Animal Health Research Case Team, Sekota Dry-land Agricultural Research Center, Sekota, Ethiopia; b Department of Veterinary Epidemiology and Public Health, College of Veterinary Medicine and Animal Sciences, University of Gondar, Gondar, Ethiopia; c Animal Health Research Case Team, Sirinka Agricultural Research Center, Sirinka, Ethiopia; d Department of Veterinary Laboratory Technology, Debre Markos University, P O Box 269, Debre Markos, Ethiopia; e International Livestock Research Institute, Addis Ababa, Ethiopia

**Keywords:** Amhara region, Attitude, Ethiopia, Knowledge, Practice, Rabies

## Abstract

**Background:**

Rabies is an important viral zoonotic disease with high fatality rate and economic losses. The impact of rabies is considerably high in Asia and Africa. The study was designed to assess the community’s rabies knowledge, attitude and practice (KAP) and their determinants in Amhara region, Ethiopia.

**Methods:**

The study was done based on a questionnaire survey of 899 participants in towns and rural districts of Amhara region. Multistage cluster sampling procedure was used to select participants. Quantitative score was generated for KAP and the scores were dichotomized as adequate and inadequate knowledge, desirable and undesirable attitude, and good and poor practice. Descriptive statics and mixed effect logistic regression considering kebele and villages as a random effect was used to see the association of predictor variables towards adequate knowledge, desirable attitude and good practice.

**Results:**

About 61%, 72% and 45% of the respondents have adequate knowledge, desirable attitude and good practice scores, respectively. Almost all (99%) of the respondents rightly claimed that rabies is transmitted through bite of dogs. Majority of the participants (76.8%) strongly agree that rabies is an important zoonotic disease that can threaten the lives of humans and animals. Only 8% of the respondents had a practice of washing and rinsing dog bite wounds for the prevention of rabies. Female participants were less likely to have adequate knowledge than males (OR = 0.58, 95% CI = 0.35–0.96). Respondents in urban areas were more likely to have desirable attitude than rural residents (OR = 12.4, CI = 1.38–11.67).

**Conclusion:**

The study showed that participants have good knowledge and attitude towards rabies but poor rabies prevention and control practices. The community public health education should focus on translation of these good knowledge and favorable attitude into practices that effectively reduces rabies burden.

## 1 Introduction

Rabies is one of the most deadly infectious diseases, with a high case fatality rate extending to 100% [1]. It is caused by rabies virus, a member of genus *Lyssa virus* in *Rhabdo viridae* family. It is clinically characterized by acute encephalitis or meningoencephalitis [2].

Globally, the annual impact of rabies is quantified as a cause of the death of 59,000 people, 3.7 million disability adjusted life years (DALYs) and loss of $8.6 billion due to premature death and costs for post exposure treatment. Rabies is a neglected and underreported disease of low-income countries where the majority of economic losses and human deaths associated with rabies are recorded [3, 4]. Domestic dog bites are the predominant source of infection to humans; 99% of human cases are originated from dog bite [5].

Rabies is highly prevalent in Ethiopia; reports indicate that it is the cause for the death of 1.6–2.33 persons per 100,000 population [6, 7]. It is a reportable disease both in human health and veterinary sector. The country has limited laboratory capacity and human rabies management. The presence of large number of dogs and their wider use as home pets in towns and as an important guard for livestock in rural areas of the country makes the disease to have wider spread and importance.

Vaccination of dogs at risk and treatment of humans with pre- and post-exposure vaccine can readily reduce diseases in humans [8]. There are problems associated with the use of post-exposure prophylaxis (PEP) in developing countries; a study in Senegal, for example, indicates even with good public awareness on its dangerous and fatal nature of rabies; only half of the patients completed the full schedule of PEP [9]. This indicates the need for repeated awareness creation about preventive measures of the disease and cautions to be taken on the appropriate time to seek treatment options and the need to complete full scheme of the treatment.

A study in South Gondar, Ethiopia, indicated low public awareness hindered the use of rabies preventive measures [10]. Some rabies knowledge, attitude and practice (KAP) studies are available in Ethiopia [11, 12, 13]. These studies showed that rabies is familiar for most members of the community but have gaps in knowledge and practice in terms of its prevention and control. However, these studies were limited to small geographic areas (mostly to a single district) and only describing the proportion of respondents in terms of certain rabies related knowledge and practices. The current study was conducted to comprehensively assess community Rabies KAP and their drivers in Amhara region.

## 2 Methods

### 2.1 Study area

**Figure 1:**
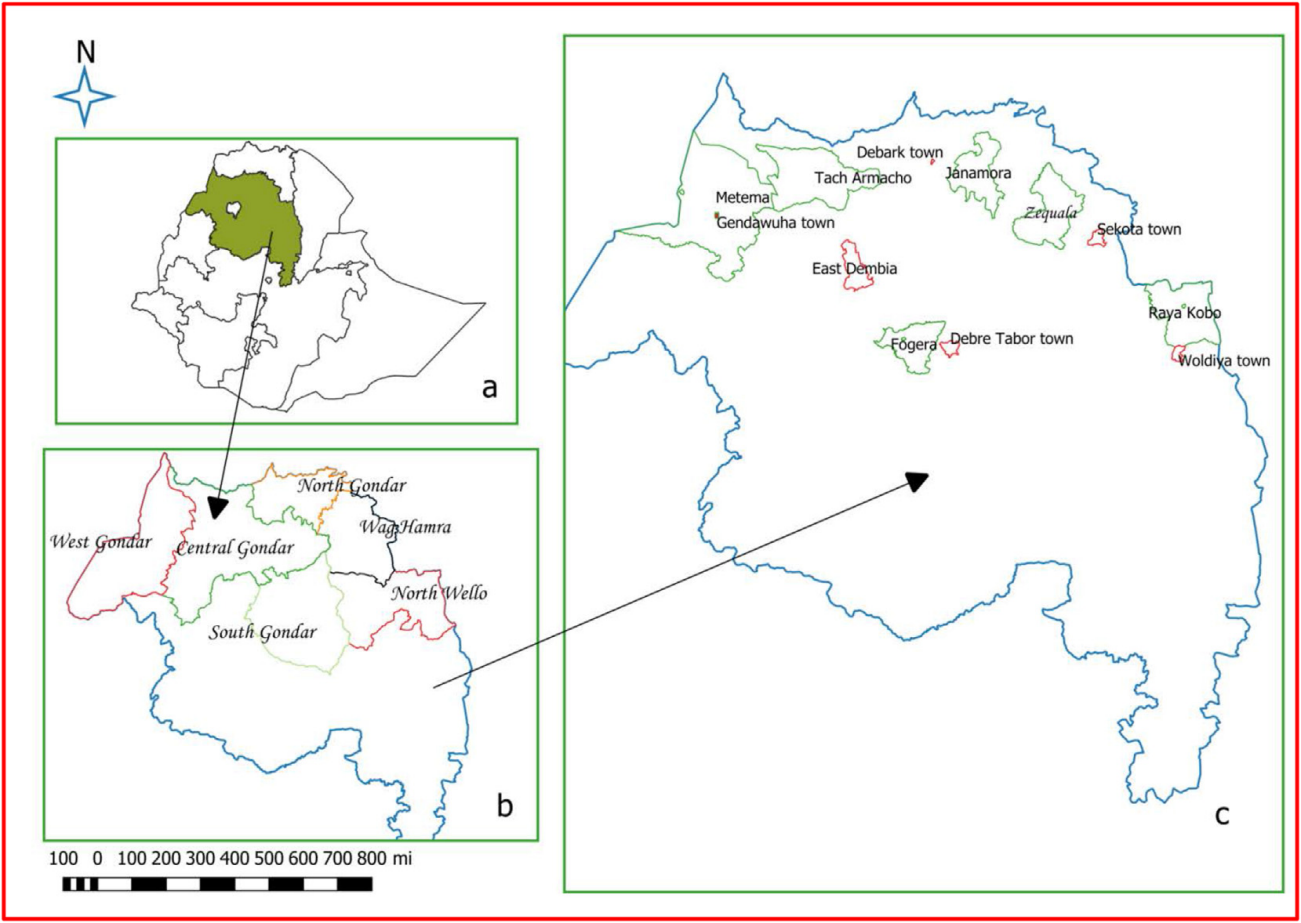
Map of the study area showing the Amara region within Ethiopia (a), and study zones (b) and districts (c) within Amhara region.

The study was done in Amhara region of Ethiopia, which is located between 9° 20 and 14° 20 and 36° 20 and 40° 20 latitude and longitude, respectively. The region is divided in to twelve administrative zones and has more than 21 million human population. About 90.85% of the population lives in rural areas. Six administrative zones and 12 districts (6 rural and 6 urban); Central Gondar (Tach Armachiho and Koladiba town), North Gondar (Janamora and Debark town), West Gondar (Metema and Gendawuha town), South Gondar (Fogera and Debretabor town), North Wollo (Raya Kobo and Woldia town) and Waghemira (Zequala and Sekota town) were included in the study (Figure 1).

The dog population in the region is not clearly known. A recent study in the region indicated a presence of large number of owned and free roaming dogs in the region [14]. The same study also reported that on average there were 1.3 dogs per dog-owned household and one in every 13 households owned dogs.

### 2.2 Study design and data collection

The study was a cross sectional study in which selected individuals from the community were interviewed once during December 2020 to June 2021 using a structured questionnaire. The questionnaire was designed to collect quantitative data on participants' knowledge, attitude and practice related to rabies and variables that could affect knowledge, attitude and practice (Supplementary material).

The questionnaire contains questions about knowledge, attitude and practices, and potential socio-demographic factors that could affect them. The questionnaire was developed based on comprehensive rabies-related literature reviews and discussion among researchers. It contains total of 55 questions which were categorized in to four: socioeconomic characteristics (18), knowledge (20), attitude (7), and practices (10) of respondents related to rabies. In knowledge and practice questions, respondents were asked to answer ‘yes’ or ‘no’ or to choose from a list of options provided. Attitude questions were developed in bipolar Likert’s scale having five components, strongly disagree, disagree, uncertain, agree and strongly agree.

The socio-demographic questions included were respondents' sex, age, socioeconomic status, marital status, geographic background (urban/rural and agro-ecology), administrative zones, educational status, dog and livestock ownership. Age class of the respondents was classified to three groups as less than 30 years, 30–45 years and greater than 45 years following previous recommendations for social science research [13].

The questionnaires were pilot tested on 10 respondents and amendments were made accordingly. The pilot testing was primarily targeted to test clarity of questions; additionally, to estimate the time needed to administer the questions without making any distress for the respondent and enough to acquire the required information. The final questionnaire was administered by face-to-face interviews using the local language (Amharic).

### 2.3 Sampling methods and sample size determination

**Figure 2:**
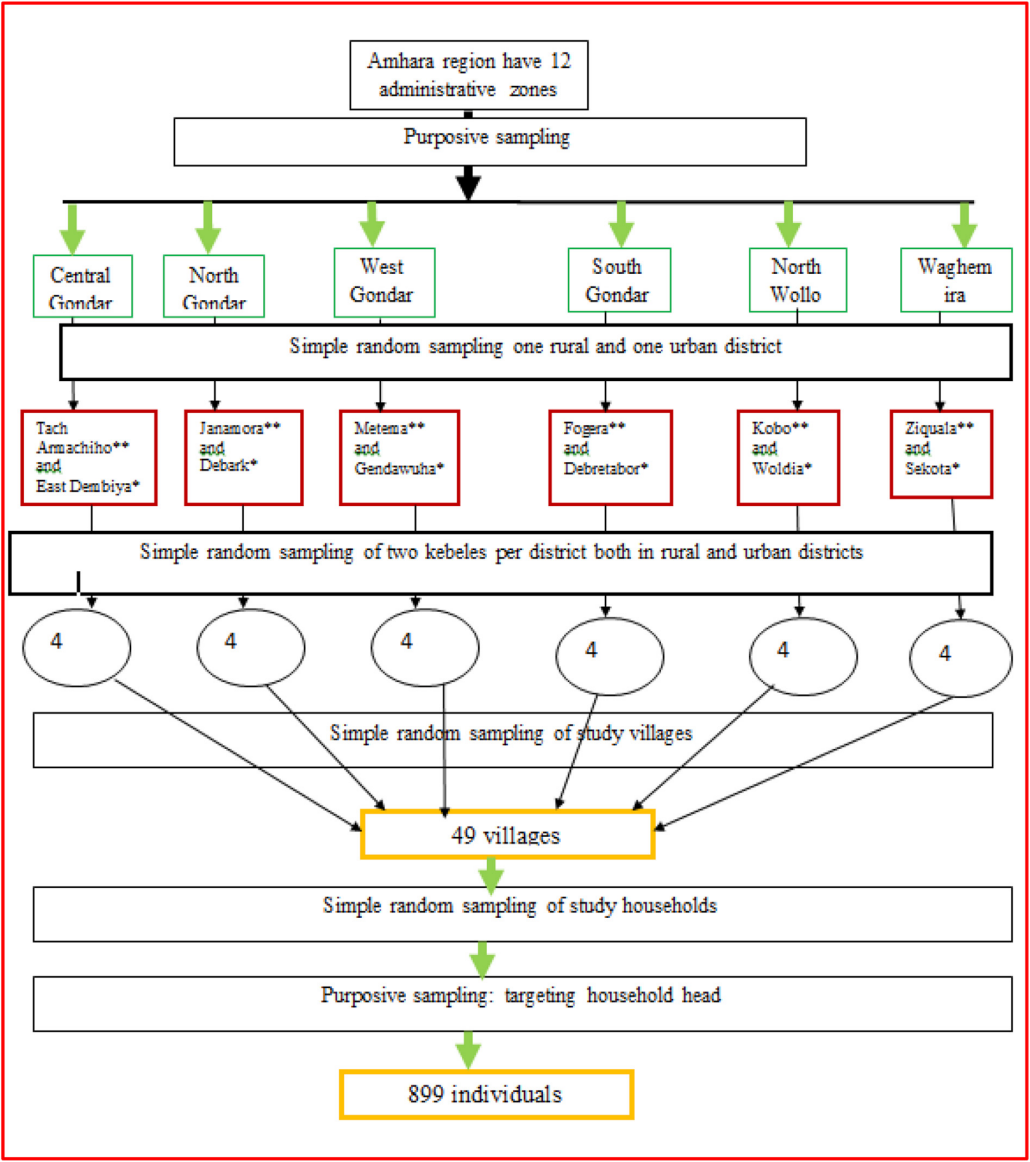
Sampling procedure for assessing knowledge, attitude and practice about rabies in Amhara region, Ethiopia. **Key:** ∗∗rural district, ∗urban district.

Multistage cluster sampling was used to select study participants. First, administrative zones were selected purposively based on their ease of accessibility. Next districts within administrative zones kebeles within districts, villages within kebeles and households within villages were selected randomly. List of kebeles were obtained from the district agricultural office and list of villages and households within kebeles were obtained from the kebele administrate office. Households were selected randomly using computer based random numbers. Individuals within household were selected purposively targeting household heads; however, subjects with age greater than 18 years of old were substituted when head of the household was not available. Household in the vicinity were substituted when no one is available or unwilling to participate from the targeted household. A similar sampling approach was followed in both urban and rural residences. The sampling approach and number of units selected at each level of sampling are shown in Figure 2. The sample size was distributed among the administrative zones proportional to their population sizes (Figure 2).

The required sample size was determined using Cochran’s formula for categorical data [15].

$$n=\frac{t^{2}*\left(p\right)\left(q\right)}{d^{2}}$$

where n = required sample size, t = value for selected alpha level of 0.025 in each tail = 1.96, p = estimate of proportion of an attribute present in the population, (q) = 1 − p, *d* = acceptable margin of error for mean being estimated. Based on the formula, the total number of households required for each of the urban and rural study sites was 384. But this was increased to 448 for urban and 451 for rural households (total sample size of 899) to made up for the design effect of cluster sampling and maximize precision. The sampling procedure and the sample size are shown in Figure 2.

### 2.4 Ethics statement

Informed verbal consent was obtained from the study participants and the study was approved by the Ethical Clearance Committee of College of Veterinary Medicine and Animal Sciences (CVMAS, Ethical clearance Committee ref. no. 1065/2022), University of Gondar, Gondar, Ethiopia.

### 2.5 Data management and analysis

The responses for KAP questions were given scores and dichotomized using cut off 50% of the maximum obtainable score reflecting good and poor KAP levels. The binary knowledge questions were given score of 1 when correctly answered 0 when answered incorrectly. Respondents who scored greater or equal to 50% were considered as having adequate knowledge and those who scored less 50% were considered as having inadequate knowledge. Similarly, responses for practice were scored as 1 (had good practice) and 0 (had poor practice). Respondents who have scored 50% and above were categorized as having good practice and individuals scored less than 50% as having poor practice.

The attitude questions were set in Likert scale and were scored as 1 = strongly disagree, 2 = disagree, 3 = uncertain, 4 = agree and 5 = strongly agree; or in reverse as 5 = strongly disagree, 4 = disagree, 3 = uncertain, 2 = agree and 1 = strongly agree depending on the nature of the statement. In both cases, the high numbered responses were given towards the desirable direction. A person’s attitude was determined based on the sum of the values given for questions measuring attitude and made binary variable into desirable attitude (for attitude score ≥ 50%) and undesirable attitude (for attitude score < 50%).

Socio-demographic characteristics and distribution of knowledge, attitude and practice of the participants were summarized using descriptive statistics. Mixed effect logistic regression model, considering kebele and village as random effect, was used to see the association of factors towards adequate knowledge, good preventive practice and desirable attitude. Sex, age category, educational status, residence, marital status, respondents position in the household, religion, livestock ownership, dog ownership and rabies knowledge level (for desirable attitude and good practice) were the predictor variables where associations were examined. Factors with a p-value of less than 0.25 in the univariable analysis were incorporated into the full multivariable mixed-effect logistic regression mode. In the multivariable mixed effect logistic regression, P-value < 0.05 was considered as cut off for statistical significance and adjusted odds ratio (AOR) and 95% CI were also calculated. A multi-collinearity test was done before fitting the variables into multivariable mixed effect logistic regression model to rule out a significant correlation between predictor variables. Confounding and interactions were checked for variables in the final model. Model validation was done using the standard Hosmer and Lemeshow test [16].

Statistical analysis was done using Statistical Package for Social Sciences (SPSS) version 25 (IBM Corp. in Armonk, NY) for descriptive statistics; whereas, associations of predictor variables with dependent variable were assessed using STATA version 14 software.

## 3 Results

### 3.1 Background characteristics of the respondents

Majority of respondents (74.4%) were males, 50.2% were from rural areas, and 50.9% were aged less than 30 years. More than two third of the respondents (72.1%) were married and 23% had education at college level and above. Besides, 44% of the study respondents were farmers and 47.3% of them have intermediate level of household income. Most of the respondents were Orthodox Christians. About 53.4% of the respondent owned livestock and only 23.2% had dogs (Table 1).

**Table 1:** Knowledge, attitudes and practices scores of study participants by socio-demographic variables in consideration to urban and rural settlement with the maximum score for each component is 1.00 (100%).

Variables	n (%)			Mean score					
				Knowledge (SD)		Attitude (SD)		Practice (SD)	
	Rural	Urban	Total	Rural	Urban	Rural	Urban	Rural	Urban
**Sex**									
Male	354 (78.5)	315 (70.3)	669 (74.4)	0.62 (0.11)	0.65 (0.11)	0.70 (0.10)	0.74 (0.09)	0.42 (0.18)	0.51 (0.18)
Female	97 (21.5)	133 (29.7)	230 (25.6)	0.58 (0.11)	0.58 (0.13)	0.68 (0.08)	0.72 (0.09)	0.37 (0.17)	0.49 (0.17)
**Age group**									
31–45 years	154 (34.1)	146 (32.6)	300 (33.4)	0.62 (0.10)	0.65 (0.10)	0.70 (0.09)	0.73 (0.09)	0.41 (0.12)	0.49 (0.19)
≥46 years	76 (16.85)	65 (14.5)	141 (15.7)	0.62 (0.09)	0.64 (0.08)	0.70 (0.10)	0.74 (0.08)	0.43 (0.18)	0.52 (0.2)
≤30 years	221 (49.0)	237 (52.9)	458 (50.9)	0.60 (0.11)	0.61 (0.11)	0.69 (0.10)	0.72 (0.09)	0.39 (0.18)	0.50 (0.16)
**Level of education**									
Illiterate	208 (46.1)	54 (12.1)	262 (29.1)	0.59 (0.11)	0.63 (0.09)	0.69 (0.09)	0.70 (0.07)	0.42 (0.12)	0.44 (0.19)
Can read and write	72 (16)	35 (7.8)	107 (11.9)	0.62 (0.10)	0.63 (0.11)	0.70 (0.10)	0.70 (0.07)	0.39 (0.19)	0.43 (0.21)
Primary education	97 (21.5)	89 (19.9)	186 (20.7)	0.61 (0.10)	0.61 (0.12)	0.68 (0.10)	0.73 (0.08)	0.40 (0.17)	0.49 (0.17)
Secondary education	49 (10.9)	88 (19.6)	137 (15.2)	0.65 (0.11)	0.60 (0.15)	0.70 (0.09)	0.74 (0.09)	0.36 (0.18)	0.52 (0.19)
College and above	25 (5.5)	182 (40.6)	207 (23)	0.61 (0.11)	0.65 (0.11)	0.75 (0.08)	0.75 (0.08)	0.47 (0.19)	0.53 (0.16)
**Marital status**									
Divorced	16 (3.5)	17 (3.8)	33 (3.7)	0.56 (0.12)	0.66 (0.12)	0.66 (0.10)	0.71 (0.08)	0.35 (0.12)	0.42 (0.19)
Married	350 (77.6)	298 (66.5)	648 (72.1)	0.62 (0.10)	0.64 (0.11)	0.70 (0.10)	0.74 (0.08)	0.43 (0.17)	0.51 (0.18)
Single	75 (16.6)	117 (26.1)	192 (21.4)	0.56 (0.12)	0.59 (0.14)	0.70 (0.09)	0.73 (0.08)	0.34 (0.19)	0.49 (0.18)
Widowed	10 (2.2)	16 (3.6)	26 (2.9)	0.6 (0.11)	0.59 (0.01)	0.69 (0.07)	0.70 (0.10)	0.28 (0.15)	0.46 (0.23)
**Religion**									
Orthodox	406 (90)	405 (90.4)	811 (90.2)	0.61 (0.11)	0.62 (0.12)	0.69 (0.10)	0.73 (0.09)	0.41 (0.18)	0.50 (0.18)
Muslim	44 (9.8)	42 (9.4)	86 (9.6)	0.58 (0.10)	0.63 (0.11)	0.70 (0.08)	0.75 (0.07)	0.38 (0.15)	0.54 (0.16)
Protestant	1 (0.2)	1 (0.2)	2 (0.2)	0.82 (0.00)	0.76 (0.00)	0.83	0.89	0.53 (0.00)	0.53 (0.00)
**Dog ownership**									
No	327 (72.5)	363 (81.0)	690 (76.8)	0.59 (0.11)	0.62 (0.11)	0.69 (0.09)	0.73 (0.08)	0.38 (0.17)	0.47 (0.17)
Yes	124 (27.5)	85 (19)	209 (23.2)	0.63 (0.09)	0.64 (0.11)	0.71 (0.10)	0.75 (0.09)	0.48 (0.18)	0.63 (0.18)
**Household income**									
Low	194 (43)	143 (31.9)	337 (37.5)	0.59 (0.12)	0.61 (0.13)	0.68 (0.09)	0.72 (0.09)	0.37 (0.17)	0.49 (0.19)
Intermediate	209 (46.3)	216 (48.2)	425 (47.3)	0.61 (0.11)	0.61 (0.13)	0.71 (0.09)	0.74 (0.08)	0.43 (0.18)	0.49 (0.18)
High	48 (10.6)	89 (19.9)	137 (15.2)	0.63 (0.11)	0.67 (0.12)	0.74 (0.11)	0.75 (0.08)	0.46 (0.21)	0.54 (0.16)
**Administrative zones**									
Central Gondar	78 (17.3)	83 (18.5)	161 (17.9)	0.61 (0.11)	0.61 (0.09)	0.63 (0.08)	0.74 (0.10)	0.34 (0.13)	0.56 (0.16)
North Gondar	78 (17.3)	76 (17)	154 (17.1)	0.58 (0.14)	0.53 (0.17)	0.72 (0.09)	0.71 (0.10)	0.40 (0.17)	0.40 (0.16)
South Gondar	75 (16.6)	78 (17.4)	153 (17)	0.63 (0.09)	0.64 (0.07)	0.73 (0.10)	0.73 (0.08)	0.48 (0.19)	0.52 (0.17)
West Gondar	77 (17.1)	69 (15.4)	146 (16.2)	0.62 (0.13)	0.68 (0.10)	0.74 (0.10)	0.73 (0.07)	0.44 (0.22)	0.40 (0.19)
North Wollo	76 (16.9)	71 (15.8)	147 (16.4)	0.55 (0.11)	0.61 (0.13)	0.66 (0.08)	0.74 (0.07)	0.38 (0.13)	0.52 (0.16)
Waghemira	67 (14.9)	71 (15.8)	138 (15.4)	0.63 (0.09)	0.68 (0.12)	0.70 (0.09)	0.75 (0.08)	0.40 (0.17)	0.60 (0.15)
**Agro-ecology**									
High land	78 (17.3)	154 (34.4)	232 (25.8)	0.58 (0.14)	0.58 (0.14)	0.72 (0.09)	0.72 (0.09)	0.40 (0.17)	0.46 (0.18)
Low land	297 (65.9)	69 (15.4)	366 (40.7)	0.60 (0.11)	0.68 (0.10)	0.68 (0.09)	0.73 (0.07)	0.39 (0.17)	0.40 (0.19)
Mid land	76 (16.9)	225 (50.2)	301 (33.5)	0.63 (0.09)	0.63 (0.12)	0.74 (0.09)	0.74 (0.08)	0.48 (0.19)	0.56 (0.16)
**Livestock ownership**									
No	106 (23.5)	313 (69.9)	419 (46.6)	0.59 (0.13)	0.62 (0.13)	0.70 (0.09)	0.73 (0.08)	0.36 (0.18)	0.48 (0.17)
Yes	345 (76.5)	135 (30.1)	480 (53.4)	0.61 (0.11)	0.62 (0.13)	0.70 (0.10)	0.74 (0.09)	0.42 (0.18)	0.55 (0.19)
**Overall**	**451(50.2)**	**448(49.8)**	**899(100)**	**0.60(0.12)**	**0.62(0.13)**	**0.69(0.10)**	**0.73(0.08)**	**0.41(0.18)**	**0.50(0.18)**
**Mean overall score**				**0.613(0.12)**		**0.715(0.09)**			**0.45(0.19)**

### 3.2 Mean score of knowledge, attitude and practice of respondents towards rabies

The mean score of knowledge, attitude and practice related to rabies were 61.3%, 71.5% and 45.46%, respectively. The mean score of knowledge, attitude and practice were 60%, 69.0% and 41% in rural districts and 62%, 73% and 50% in urban districts, respectively (Table 1). In general, 84.9%, 98.6% and 43% of the participants had adequate knowledge, desirable attitude, and good practice towards rabies, respectively.

### 3.3 Respondents' knowledge towards rabies

All participants knew that rabies affects dogs. About 94% of them knew rabies affects equines too and about 53% of respondents accept cats can be affected by rabies. Regarding transmission, 99.4% of respondents admit dog bite is the main means of transmission and 44.3% and 80.2% of them believe that wound licking and skin scratches, respectively were additional routes for the transmission of rabies. About 98.8% of the participants reflected that rabies causes aggressiveness, and 62.4% of them knew that most of the clinical signs associated with rabies are nervous signs (Table 2).

**Table 2:** Frequency distribution of respondents' response for knowledge questions on rabies.

Variables	No. of correct and/yes respondents (%)
The following species are susceptible host that can be affected with rabies	
Dogs	899 (100)
Human	880 (98.9)
Equine	846 (94.1)
Cattle	824 (91.7)
Sheep (goat)	782 (87.0)
Fox	720 (80.1)
Cat	480 (53.4)
Hyena	256 (28.5)
Pig	120 (13.3)
The following is important means of rabies transmission	
Bite	894 (99.4)
Skin scratch	721 (80.2)
Wound licking	398 (44.3)
Other than all of the above	5 (0.6)
The following species can be source of rabies for human other than dogs	
No other sources than dog	141 (15.7)
Equine	758 (84.3)
Fox	497 (55.3)
Cat	251 (27.9)
Hyena	114 (12.7)
The following body parts of the animals involved in rabies disease	
Brain	561 (62.4)
Local infection at the site of bite	83 (9.3)
I don’t know	338 (37.6)
The following is the clinical signs of rabies disease in dog and or human	
Aggressiveness	888 (98.8)
Protruding of the tongue	873 (97.1)
Profuse salivation	821 (91.3)
Dropping of tail	717 (79.8)
Dropping of head and neck	566 (63.0)
Eating of abnormal items	370 (41.2)
Hydrophobia	171 (19.0)
Difficulty in swallowing	61 (6.8)
Change in sound	9 (1.0)
Photophobia	03 (0.3)
I don’t know	11 (1.2)
Stray dogs are more involved in rabies transmission than owned dogs	864 (96.1)
Dog wandering out of the community could be a source of infection	799 (88.9)
Susceptibility to rabies is different across age and sex groups	605 (67.3)
There is difference in exposure potential to rabies between different occupation groups	665 (74.0)
Have you ever seen rabid animal and define it was rabies?	534 (59.4)
Have you ever seen rabid human and define it was rabies?	457 (50.8)
Can rabid person recover without treatment (traditional or modern)	836 (93.0)
Do you know the availability of traditional treatment for rabies	598 (66.5)
Do you know the availability of post exposure prophylaxis (PEP) as a treatment for rabies?	806 (89.7)
Modern treatment options shall be preferred over traditional options for rabies exposure, as there is no evidence confirming their effectiveness	575 (63.7)
Can rabies be treated after development of clinical sign?	613 (68.2)
Is rabies a preventable disease?	753 (83.8)
Does vaccination for human works after rabid dog bite?	452 (50.3)
Do you know the availability of vaccines for the prevention of rabies in dog?	622 (69.2)
Do you know the interval for vaccination of dogs?	177 (19.7)

### 3.4 Attitudes of participants towards rabies

Most of the participants (76.8%) strongly agree while 2.6% strongly disagree that rabies is an important zoonotic disease. About 45% of the participants believed that consumption of meat from rabid animal could be a source of infection to humans whereas 8.1% and 4.1% of the respondents strongly accepts that, respectively, consumption of meat and inhalation of burns from dead animals can prevent rabies infection. About 42.3% of the respondents were uncertain about the effectiveness of dog vaccination against rabies (Table 3).

**Table 3:** Frequency distributions of respondents' attitude towards rabies.

Questions	Response categories n (%)				
	Strongly disagree	Disagree	Uncertain	Agree	Strongly agree
Rabies is an important zoonotic disease	23 (2.6)	5 (0.6)	13 (1.4)	168 (18.7)	690 (76.8)
Consumption of meat from animal died of rabies is a source of infection	277 (30.8)	127 (14.1)	88 (9.8)	186 (20.7)	221 (24.6)
Consumption of meat from animal died of rabies is preventive	304 (33.8)	217 (24.1)	161 (17.9)	144 (16.0)	73 (8.1)
Inhalation of meat burn from animal died of rabies is preventive	261 (29.0)	197 (21.9)	330 (36.7)	74 (8.2)	37 (4.1)
Crossing river within 40 days of bite inactivate the effect of PEP/traditional treatment	202 (22.5)	175 (19.5)	215 (23.9)	207 (23.0)	100 (11.1)
I always contact health professional after dog bite in fear of rabies	45 (5.0)	236 (26.3)	54 (6.0)	361 (40.2)	203 (22.6)
Vaccination for dogs is effective in preventing rabies	27 (3.0)	91 (10.1)	380 (42.3)	307 (34.1)	94 (10.5)

### 3.5 Practice of participants towards rabies

Majority of dog owners do not use indoor management and 16.6% of the participants do nothing in fear of rabies during a dog bite unless the wound is severe (Table 4).

**Table 4:** Frequency distribution of respondents' practice towards rabies.

Variables	No. ​of ​respondents with ​good ​practice and/responding yes (%)
Do you use indoor management for your dog?	76 (36.4)
Have you ever practiced traditional treatment in favor of modern vaccination against rabies?	344 (38.3)
Do you practice the following measures when a dog bite is encountered	
Do nothing	149 (16.6)
seek health professional help	750 (83.4)
Tie the dog and follow for signs	356 (39.6)
Wash and rinse the site of bite	72 (8.0)
Do you take the following measures on animal bitten by a rabid dog?	
Slaughter and consumption of meat	673 (74.9)
seek advice from animal health expert	98 (11.0)
Have you ever vaccinated your dog?	109 (52.1)
Do you vaccinate your dog every year?	78 (37.3)
Do you practice the following measures when a dog is suspected of having rabies?	
Immediate killing of dogs	221 (24.6)
Tie to see overt clinical signs	678 (75.4)
Inform the veterinarian in charge	274 (30.5)
Do you avoid contact from unknown or wild animals in fear of rabies?	672 (74.7)
Do you take any safety measure when you are caring rabies suspected patient	611 (68.0)
Do you practice the following safety measures?	
I do use no safety measures	288 (32.0)
Protect from bite	610 (67.9)
Avoid contact with saliva	554 (61.6)

### 3.6 Factors associated with participant’s knowledge, attitude and practice towards rabies

Sex, the respondent’s position within the household, age category, educational level, marital status and livestock ownership showed a significant association at a p-value of 0.25 and are incorporated in multivariable mixed effect logistic regression. In the multivariable mixed-effect logistic regression model (kebele and village taken as random effect), all of these variables except livestock ownership were associated with knowledge level of the respondents (Table 5).

**Table 5:** Multivariable mixed effect logistic regression predicting rabies related knowledge among community members of Amhara region.

Variables	Category	COR (95% CI)	P-value	AOR (95% CI)	p-value
Sex	Male	Ref		Ref.	
	Female	0.40 (0.26–0.62)	<0.001	0.58 (0.35–0.96)	0.035
the respondent’s position within the household	Other	Ref.		Ref	
	Household head	3.16 (2.01–4.97)	<0.001	1.99 (1.19–3.35)	0.009
Age category	≤30 years			Ref	
	31–45 years	2.45 (1.53–3.92)	<0.001	1.83 (1.11–3.03)	0.018
	≥46 years	2.17 (1.29–3.66)	0.004	1.51 (0.86–2.65)	0.148
Education level	Illiterate			Ref.	
	Can read and write	1.50 (0.77–2.92)	0.228	1.40 (0.70–2.80)	0.343
	Primary education	1.55 (0.89–2.73)	0.124	1.96 (1.05–3.65)	0.033
	Secondary education	1.63 (0.86–3.10)	0.134	4.30 (2.02–9.16)	<0.001
	College and above	2.79 (1.43–5.43)	0.003	5.60 (2.64–11.89)	<0.001
Marital status	Single			Ref.	
	Married	2.56 (1.64–3.99)	<0.001	1.94 (1.08–3.49)	0.027
	Divorced	1.25 (0.48–3.24)	0.651	1.35 (0.44–4.22)	0.583
	Widowed	1.32 (0.43–4.07)	0.634	1.58 (0.41–6.00)	0.505
Livestock ownership	No	Ref.			
	Yes	1.35 (0.86–2.11)	0.192		

COR = crude odds ratio, AOR = adjusted odds ratio, Ref. = reference.

The attitudes multivariable mixed effect logistic regression model showed residence was the only explanatory variable associated with the respondents' attitude towards rabies. Respondents in urban areas were 12.40 (95%CI: 1.38–111.67) times more likely to have a desirable attitude towards rabies than rural residents.

The multivariable mixed effect logistic regression model of rabies related practice of respondents showed sex, residence, dog ownership and rabies knowledge level were significant predictors of practice (Table 6).

**Table 6:** Multivariable mixed effect logistic regression model that shows factors associated with participants' practice towards rabies in Amhara region.

Variable	Category	COR (95% CI)	P-value	AOR (95% CI)	p-value
Sex	Male	Ref.			
	Female	0.65 (0.49–0.94)	0.024		
Residence	Rural	Ref.			
	Urban	2.68 (1.21–5.90)	0.015	2.70 (1.18–6.19)	0.019
Education level	Illiterate	Ref.		Ref.	
	Can read and write	0.90 (0.52–1.57)	0.717	0.69 (0.38–1.22)	0.192
	Primary education	1.40 (0.88–2.21)	0.153	1.26 (0.80–1.91)	0.330
	Secondary education	1.64 (0.98–2.74)	0.062	1.51 (0.85–2.67)	0.157
	College and above	2.63 (1.60–4.32)	<0.001	2.55 (1.47–4.42)	0.001
Marital status	Single	Ref.			
	Married	1.21 (0.82–1.78)	0.335		
	Divorced	0.54 (0.22–1.35)	0.189		
	Widowed	0.41 (0.14–1.14)	0.088		
Dog ownership	No	Ref.		Ref.	
	Yes	3.87 (2.59–5.79)	<0.001	4.09 (2.67–6.30)	<0.001
Livestock ownership	No	Ref.			
	Yes	1.61 (1.10–2.35)	0.014		
Rabies knowledge level	Inadequate			Ref.	
	Adequate	2.26 (1.40–3.63)	0.001	1.94 (1.16–3.23)	0.011

COR = crude odds ratio, AOR = adjusted odds ratio, Ref. = reference.

## 4 Discussion

The study presents findings of KAP on rabies in urban and rural areas of Amhara region, Ethiopia. It illustrates that most of the respondents had good knowledge and a desirable attitude towards rabies, whereas their preventive practice was poor. About 84.9% of the respondents have adequate knowledge. The result in the current study is consistent with reports in previous studies by Ali et al. [17] and Digafe et al. [12] who reported 83% and 90.8% of having adequate from Addis Ababa and Gondar, respectively. However, it was higher than 64.1% reported from Bahir Dar by Guadu et al. [11] and 56.1% from Mekelle by Hagos et al. [18]. The result in the current study is also roughly similar to reports from other endemic countries such as Indonesia (82.6%) [19], Guatemala (82%) [20] and Tanzania (96%) [21]. The result shows the disease is endemic in the study area and there is adequate knowledge in community.

In the current study about 98.6% of the respondents have a desirable attitude towards rabies. The result is much higher compared to reports in previous studies where only 56.2% of the respondents had a positive attitude towards rabies [18]. This can indicate that the community has a better chance of implementing control measures if the concerned bodies are doing their best in this regard. However, observations in this study showed that only 43% of the respondents had good practices towards rabies. This is much lower than previous reports in Ethiopia such as 61.3% in Mekelle town [18] and also in other African country, Nigeria (74%) [22]. The lower preventive practice score in the current study might be associated with the involvement of participants from rural areas whereas in previous studies (e.g. in Mekelle, Ethiopia), participants were drawn from urban areas. Previous studies reported showed that pet care practices are better in urban than rural areas [23].

Findings of the current study showed that respondents in urban areas had considerably higher scores of practice than rural respondents. The result is consistent with previous studies of KAP on public health important issues conducted in Ethiopia [24, 25] and other countries including China [26], India [27]. This is not surprising as most public health education and accessibility to health infrastructures are concentrated in urban than rural areas and this allows them to have better preventive practice measures. Urban residents have greater resources, higher density and better infrastructures to have better access to health service and education [23, 28].

Almost all (98.9%) of the respondents know that the main source of rabies in humans is dogs. This is in agreement with the WHO report that 99% of rabies in humans is from rabid dog bites [29]. Non negligible numbers (15.7%) of respondents believe that species other than a dog can never be a threat for human rabies. This is not consistent with the epidemiology of the disease as it can also be contracted from other rabies susceptible species [30]. This suggests the need for creating awareness about the potential sources of rabies to humans other than dogs.

About 98.9% of the respondents knew the clinical signs of rabies in human and animals. They revealed that neurological signs are the predominant clinical manifestation in dogs. A similar finding was reported in Rwanda and Chad indicating most of the respondents know the clinical manifestations of rabies [31, 32]. There is a perception from the respondents that, 62.4% of them knew the virus could reach the central nervous system (brain) while the remaining believed the nervous sign in humans is due to the formation of puppies and their disturbance in the abdomen. This perception might be associated with the manifestation of abdominal pain in the early course of rabies illness as reported by Ayatollahi et al. [33]; however, the formation of puppies in the abdominal cavity is obviously a misconception that has no scientific plausibility. This widespread misconception needs to be addressed properly through targeted community health education.

Most of the respondents knew that rabies is a fatal disease and cannot cure without treatment. Additionally, 89.2% of them knew the availability of PEP as a treatment for rabies exposed individuals. This favorable knowledge is helpful to implement the recommendations that any individual with a bite suspected of having rabies should seek medical attention as quickly as possible [34]. However, 63.7% of the respondents accept that traditional treatments are more effective, but this is problematic as the effectiveness of most of traditional treatments is not known. Generally, the community has good knowledge of the availability of modern medical treatment and the fatal nature of the disease if left untreated. However, this favorable knowledge is masked by their treatment options which incline to traditional treatments and there is a need to create awareness among the public to drive their demand for modern medical options.

About 31.8% of respondents believe that rabies can be treated after the onset of clinical signs. This is not consistent with the facts that ones the clinical signs are seen there is no way for recovery [3]. The respondents argue that traditional healers can diagnose the formation of puppies in the stomach of the victims due to rabid dog bites and they can treat the victims.

In this study, a considerable proportion (44.9%) of the respondents believe consumption of meat from an animal that died of rabies is a source of infection and discard to the surroundings to be eaten by wild and domestic canines. This could be taken as a good attitude to minimize risk of rabies however, it demands teaching the community about proper disposal to avoid the potential means of disease dissemination. Similar practices were reported in Ethiopia from Jimma where 65% of the respondents [35] and Tanzania where 25% of the respondents [21] used to throw the carcass in fear of rabies. The habit of throwing the carcass and allowing access to canines may result in the dissemination of the disease. In relation to this an outbreak of rabies was recorded in South Africa from consumption of the cranium of rabid Jackal [36]. On the other side, 24.1% and 12.1% of the respondents believe consumption of meat and inhalation of meat burned from food animals died of rabies is important for rabies prevention. Similar work in South Gondar, Ethiopia indicated the practice of consuming meat from rabid animals as a method of prevention [10]. Although, it can be thought that low virus dose exposure through consumption of contaminated meat would help to develop immunity against the disease, this has to be discouraged until reliable scientific evidence is available about such practices.

About 92% of the respondents do not practice immediate washing and flushing with water at the site of infection. The result is in agreement with previous studies in Ethiopia [12] and another African country in Uganda [37] where proper wound washing after a dog bite was not practiced. The WHO recommends that an immediate washing at the site of bite is the first and most important component of PEP [29]; wound washing and flushing reduces the impact of the disease by five folds [30]. The low-level wound washing practice in this study indicates the important and easily accessible portion of the PEP is missed. Therefore, the community needs detailed training in this regard.

Around 60.9% of the respondents practice an immediate killing of the dog when they encounter a bite of human. This finding is in agreement with previous reports in Ethiopia (47.7%) [34] and India (42%) [38]. However, it contradicts the recommended measure that a dog bitten a human should be tied for ten days and monitored to see overt clinical signs [39].

There is a statistically significant difference in knowledge scores between males and females, where males are more likely to have adequate knowledge than females. The result is in agreement with previous reports [11, 22, 40]. The result may not be surprising as most of the outdoor communication and knowledge sharing in Ethiopia happens between males. Respondents with a formal education background have a higher knowledge of rabies compared to illiterates. The result is consistent with previous reports where participants with higher education levels were more likely to have adequate knowledge [11, 41, 42]. Educated people could have better information access and can easily understand the impact of the disease and health education. Respondents who are single have a relatively lower knowledge than those who are married and divorced. Similar results have been reported where married individuals have a higher knowledge as compared with single participants [18, 43]. This might be associated with the opportunity to share health information between the partners. The current study shows that adult (31–45 years) respondents were more likely to have adequate knowledge than young individuals (<30 years). This is in agreement with previous a report from the North Shewa zone of Ethiopia [44]. This might be associated with the long time exposure/experience of adult individuals about the disease.

The current study shows respondents from urban areas were more likely to have a desirable attitude towards rabies than rural residents. A similar report indicated that attitudes and pet care practices relevant to limit spread of rabies were different in urban and rural areas where urban residents were more aware about the disease [23]. This might be associated with better education level of people residing in town and the higher health education services in urban areas.

Respondents with education levels of college and above were more likely to have good practice than respondents who are illiterate. This is in line with a report from Bahir Dar, Ethiopia, which indicates respondents with education level of college and above were more likely to have higher preventive practice scores than those who didn’t attend formal school learning [11]. This couldn’t be surprising as educated people can access better information and act on means of preventive practices. This could be a timely reminder for public health education service providers to make their prior focus on individuals who didn’t attend formal school learning.

Furthermore, respondents who own a dog are more likely to have a good practice score than those who have no dog. A similar report from India indicates dog ownership has a positive contribution in performing preventive practice measures [27] and they are more cooperative in performing rabies control measures [23]. This might be attributed to the expected risks associated with owning a dog so a higher care practice wouldn’t be surprising.

## 5 Conclusion

This study revealed that there is a good level of knowledge and attitude components of KAP whereas the level of preventive practice was low in Amhara region of Ethiopia. The average score of knowledge, attitude and practice was 61.3%, 71.5% and 45% respectively. Those involved in health education should address the problem associated with the widespread traditional anti rabies pre and post treatment practices until it is scientifically investigated. Attention should be given by public health authority to create awareness in the community about simple but important rabies preventive measures such as immediate washing of wound following dog bite and quarantine of the biting dog. Females, community members with low level education and residents in rural areas should get a high focus in public health education towards rabies.

## Declarations

### Author contribution statement

Adane Bahiru: Conceived and designed the experiments; performed the experiments; analyzed and interpreted the data; Wrote the paper.

Wassie Molla, Wudu Temesgen Jemberu, Sefinew Alemu Mekonnen: Conceived and designed the experiments; analyzed and interpreted the data; wrote the paper.

Liuel Yizengaw: Performed the experiments.

### Funding statement

This work was supported by University of Gondar and Amhara Region Agricultural Research Institute (CVMAS/13/313/2013).

### Data availability statement

Data will be made available on request.

### Declaration of interest statement

The authors declare no conflict of interest.

### Additional information

No additional information is available for this paper.
