# Supplementary material for: Knowledge, attitude and practice related to rabies among residents of Amhara region, Ethiopia
Source: Heliyon. 2022 Nov 3;8(11):e11366. doi: 10.1016/j.heliyon.2022.e11366 (PMC9649959; doi:10.1016/j.heliyon.2022.e11366)
Supplement: Supplementary material [file mmc1.docx]

## ****Supplementary material****

## Questionnaires for the assessment of Knowledge, Attitude and Practice (KAP) Related to Rabies among Residents of Amhara Region, Ethiopia

I am ………... From University of Gondar who is doing research on the assessment of knowledge, attitude and practice of respondents towards rabies. Your contribution for achieving the objective of the research is highly valuable, and incase if you don’t want to participate in the interview process you are not obligated to finalize the interview.

Do you agree to participate in this survey (note that you can withdraw from the survey at any time)? Yes [ ] No [ ]

Thank you in advance for your cooperation!!

Enumerator’s name _________________ Signature _________ date __________

**Part I: General information of the Study area**

1. Administrative zone __________________
2. District ____________
3. Kebele___________
4. Village ___________
5. Geo-reference of the village: longitude ______ latitude ______altitude______
6. Agro-ecology: high land _________ mid-land___________ low land________

**Part II: Basic Information of the Respondent**

1. Name of the respondent _____________________ Age __________ sex __________
2. are you the head of the house hold (tick 🗸choice) yes [ ] no [ ]
3. educational level of the respondent (tick 🗸choice): illiterate [ ] can read and write [ ] primary education [ ] secondary education [ ] college and above [ ]
4. geographic back ground (tick 🗸choice): urban [ ] rural [ ]
5. marital status (tick 🗸choice): married [ ] single [ ] divorced [ ] widowed [ ]
6. occupation (tick 🗸choice): farmer [ ] merchant [ ] civil servant [ ] daily laborer [ ] others (specify) [ ]
7. How much is the annual income of the household (in ETB)?
8. Religion: Orthodox [ ] Muslim [ ] Protestant [ ] others [ ]
9. For how long do you stay in the area? (tick 🗸choice): 1-5 years [ ] 6-10 years [ ] >10 years [ ]
10. What is the total size of the household? _____ male ____ female ____ number of dependents ____
11. Do you own dogs? (tick 🗸choice): yes [ ] no [ ]

Q12 and 13 will be answered if you do not own dogs

1. If your answer for Q12 is no, what was your reason? (Tick **🗸** choice)
2. Do not like [ ]
3. Have no space [ ]
4. Require much time/money [ ]
5. Fear of zonootic disease [ ]
6. Incapable of managing them [ ]
7. Other reasons specify _____________
8. Have you planned to own dogs for the future? (Tick **🗸** choice): yes [ ] no [ ]
9. What is the role of dog in your house hold? (Tick **🗸** choice)
10. Recreation [ ]
11. Home guard [ ]
12. Field guard of small ruminants/crop [ ]
13. Not applicable (for those who do not own and no plan to own) [ ]
14. Do you have domestic livestock? (Tick **🗸** choice): yes [ ] no [ ]
15. If your answer for Q16 is yes, what are they? _________, __________, ________,________, __________,_________
16. Have you ever heard of rabies? (Tick **🗸** choice): yes [ ] no [ ]
17. In which season does rabies outbreak is common in your area?

**Part III. Questionnaire for the assessment of knowledge towards rabies**

1. The following species are susceptible host that can be with rabies
2. Dog
3. Cat
4. Human
5. Sheep and goat
6. Cattle
7. Equines
8. Fox
9. Pig
10. Hyena
11. The following is important means of rabies transmission
12. Bites
13. Wound licking
14. Skin scratches
15. Do you think stray dogs are responsible for the transmission of rabies than own dog? (Tick **🗸** choice): yes [ ] (1/1) no [ ] (0/1)
16. Do you think that your dog travel outside of the nearest communities and can be a source of rabies to the family? (Tick **🗸** choice): yes [ ] (1/1) no [ ] (0/1)
17. The following species can be source of rabies for human other than dogs
18. Cat
19. Equines
20. Cat
21. Hyena
22. Do you think susceptibility to rabies is different across different age groups and between sexes of humans? (Tick **🗸** choice): yes [ ] (0/1) no [ ] (1/1)
23. The following is the clinical signs of rabies disease in dog and or human
24. Aggressiveness
25. Protruding of the tongue
26. Profuse salivation
27. Dropping of tail and Twisting of the tail back between legs
28. Dropping of head and neck
29. Eating abnormal items
30. Hydrophobia
31. Difficulty in swallowing/reduced appetite
32. Change in sound
33. Leave the environment
34. Have you ever seen rabid animals and define it was rabies? (Tick **🗸** choice): yes [ ] (1/1) no [ ] (0/1) I heard [ ] (0/1)
35. Have you ever seen rabid human and define it was rabies? (Tick **🗸** choice): yes [ ] (0/1) no [ ] (1/1) I heard [ ] (0/1)
36. The following the body parts of animals involved are in rabies disease
37. Brain
38. Local infection at the site of bite
39. Rabid man can recover without treatment (traditional or modern)
40. No [ ] (1/1)
41. Yes [ ] (0/1)
42. Do you think there is a difference in exposure potential between differences in occupation, age and gender? (Tick **🗸** choice): yes [ ] (1/1) no [ ] (0/1)
43. Do you know potential traditional treatment centers for rabies? (Tick **🗸** choice): yes [ ] (1/1) no [ ] (0/1)
44. Do you know the availability of potential modern treatment centers for rabies? (Tick **🗸** choice): yes [ ] (1/1) no [ ] (0/1)
45. Modern treatment options shall be preferred over traditional for rabies exposure, as there is no evidence confirming their effectiveness
46. Yes [ ] (1/1)
47. No [ ] (0/1)
48. Do you think rabies vaccination for human works after rabid dog bite? (Tick **🗸** choice): yes [ ] (1/1) no [ ] (0/1)
49. Can it be treated after the onset of clinical sign? (Tick **🗸** choice): yes [ ] (0/1) no [ ] (1/1)
50. Is rabies a preventable disease? (Tick **🗸** choice): yes [ ] (1/1) no [ ] (0/1)
51. Do you know the availability of vaccines for the prevention of rabies in dog? (Tick **🗸** choice): yes [ ] (1/1) no [ ] (0/1)
52. Do you know the interval for vaccination of dogs? (Tick **🗸** choice): yes [ ] (1/1) no [ ] (0/1)

**Part IV. Questionnaire for measuring attitude of the participants**

1. Rabies is an important zoonotic disease.
2. Strongly disagree
3. Disagree
4. Uncertain
5. Agree
6. Strongly agree
7. Consumption of meat of an animal dead of rabies can be a cause for the transmission of rabies.
8. Strongly disagree
9. Disagree
10. Uncertain
11. Agree
12. Strongly agree
13. Consumption of meat of an animal dead of rabies is preventive against rabies.
14. Strongly disagree
15. Disagree
16. Uncertain
17. Agree
18. Strongly agree
19. Inhalation of the burn of an animal dead of rabies is preventive against rabies.
20. Strongly disagree
21. Disagree
22. Uncertain
23. Agree
24. Strongly agree
25. Crossing the river before 40 days will make the traditional and or modern medicine ineffective.
26. Strongly disagree
27. Disagree
28. Uncertain
29. Agree
30. Strongly agree
31. If I have bitten by a dog, I should always consult health professionals immediately.
32. Strongly disagree
33. Disagree
34. Uncertain
35. Agree
36. Strongly agree
37. How satisfied are you with the vaccination campaign provided for the community dogs?
38. Extremely dissatisfied
39. Dissatisfied
40. Neither dissatisfied nor satisfied
41. Satisfied
42. Extremely satisfied

**Part V: questionnaire for measuring practices of the participant**

1. Do you use indoor management for your dog? (Tick **🗸** choice): yes [ ] (1/1) no [ ] (0/1)
2. Have you ever practiced traditional vaccination in favor of medical vaccination against rabies? (Tick **🗸** choice): yes [ ] (0/1) no [ ] (1/1)
3. Do you practice the following measures when a dog bite is encountered?
4. Immediate wash and rinse with water at the site of bite
5. Tie the dog and follow for 10 days
6. Search for health professional for assistance
7. Do you take the following measures on animal bitten by a rabid dog?
8. Search a veterinarian for decision
9. Slaughter and consumption of meat
10. Have you ever vaccinated your dog? (Tick **🗸** choice): yes [ ] (1/1) no [ ] (0/1)
11. Do you vaccinate your dog every year? (Tick **🗸** choice): yes [ ] (1/1) no [ ] (0/1)
12. Do you practice the following measures you take on a dog suspected of having rabies?
13. Tie and follow for the development of clinical signs
14. Immediate killing of dogs
15. Inform the veterinarian/health in charge
16. Do you avoid contact from unknown or wild animals? (Tick **🗸** choice): yes [ ] (1/1) no [ ] (0/1)
17. Do you take any safety measure when you are caring rabies suspected patient (even for the future) (Tick **🗸** choice): yes [ ] (1/1) no [ ] (0/1)
18. Do you practice the following safety measures as fear for rabies?
19. Avoid contact with saliva
20. Protect yourself from bite
